# Efficiency Analysis of Powertrain for Internal Combustion Engine and Hydrogen Fuel Cell Tractor According to Agricultural Operations

**DOI:** 10.3390/s24175494

**Published:** 2024-08-24

**Authors:** Hyeon-Ho Jeon, Seung-Yun Baek, Seung-Min Baek, Jang-Young Choi, Yeon-Soo Kim, Wan-Soo Kim, Yong-Joo Kim

**Affiliations:** 1 Department of Smart Agriculture Systems, Chungnam National University, Daejeon 34134, Republic of Korea; jhh0428@o.cnu.ac.kr (H.-H.J.); kelpie0037@cnu.ac.kr (S.-Y.B.); baeksm@cnu.ac.kr (S.-M.B.); 2 Department of Electrical Engineering, Chungnam National University, Daejeon 34134, Republic of Korea; choi_jy@cnu.ac.kr; 3 Department of Bio-Industrial Machinery Engineering, Pusan National University, Miryang 50463, Republic of Korea; yskim23@pusan.ac.kr; 4 Department of Bio-Industrial Machinery Engineering, Kyungpook National University, Daegu 41566, Republic of Korea; wansoo.kim@knu.ac.kr; 5 Upland Field Machinery Research Center, Kyungpook National University, Daegu 41566, Republic of Korea; 6 Department of Biosystems Machinery Engineering, Chungnam National University, Daejeon 34134, Republic of Korea

**Keywords:** hydrogen fuel cell tractor, powertrain, efficiency analysis, load measurement system, agricultural operation

## Abstract

As interest in eco-friendly work vehicles grows, research on the powertrains of eco-friendly tractors has increased, including research on the development of eco-friendly vehicles (tractors) using hydrogen fuel cell power packs and batteries. However, batteries require a long time to charge and have a short operating time due to their low energy efficiency compared with hydrogen fuel cell power packs. Therefore, recent studies have focused on the development of tractors using hydrogen fuel cell power packs; however, there is a lack of research on powertrain performance analysis considering actual working conditions. To evaluate vehicle performance, an actual load measurement during agricultural operation must be conducted. The objective of this study was to conduct an efficiency analysis of powertrains according to their power source using data measured during agricultural operations. A performance evaluation with respect to efficiency was performed through comparison and an analysis with internal combustion engine tractors of the same level. The specifications of the transmission for hydrogen fuel cell and engine tractors were used in this study. The power loss and efficiency of the transmission were calculated using ISO 14179-1 equations, as shown below. Plow tillage and rotary tillage operations were conducted for data measurement. The measurement system consists of four components. The engine data load measurement was calculated using the vehicle’s controller area network (CAN) data, the axle load was measured using an axle torque meter and proximity sensors, and fuel consumption was measured using the sensor installed on the fuel line. The calculated capacities, considering the engine’s fuel efficiency for plow and rotary tillage operations, were 131.2 and 175.1 kWh, respectively. The capacity of the required power, considering the powertrain’s efficiency for hydrogen fuel cell tractors with respect to plow and rotary tillage operations, was calculated using the efficiency of the motor, inverter, and power pack, and 51.3 and 62.9 kWh were the values obtained, respectively. Considering these factors, the engine exhibited an efficiency of about 47.9% compared with the power pack in the case of plow tillage operations, and the engine exhibited an efficiency of about 29.3% in the case of rotary tillage operations. A hydrogen fuel cell tractor is considered suitable for high-efficiency and eco-friendly vehicles because it can operate on eco-friendly power sources while providing the advantages of a motor.

## 1. Introduction

Global warming is caused, in part, by environmental pollution issues and greenhouse gas emissions from diesel engines [1]. In particular, particle mass (PM) emissions are so high that emissions from agricultural and construction equipment account for 25.9% of non-road mobile pollution sources in South Korea. Interest in the development of eco-friendly power sources that can replace existing internal combustion engines continues to increase. For example, John Deere, CNH, and Fendt have recently commercialized eco-friendly products [2,3,4] with a focus on small tractors that are being distributed in European and American markets. In Korea, eco-friendly tractors have been developed by manufacturers such as Dae-dong, LS Mtron, and TYM [5,6,7,8,9].

Related research has been conducted on hybrid systems using internal combustion engines and electric motors, and various studies have examined electrified powertrains that use batteries and hydrogen fuel cell power packs [10,11,12,13,14,15,16]. Francesco [17] researched design optimization for electrical tractors by conducting performance evaluations of hybrid systems (engines and electrical motors) using measurement systems. Baek [18] conducted research about a power system for the 120 kW tractor using independent motors on four axles. Baek predicted the time available for agricultural operation through a battery SOC (State of Charge) analysis using measured load data. Yikun [19] researched a powertrain system with two motors. Research on eco-friendly tractors has focused on control and drive systems that can select the number of drive motors depending on the load level, and on optimizing power systems based on workloads.

As previously mentioned, research on the powertrains and systems of eco-friendly tractors is being conducted in various forms. Eco-friendly tractors consist of a system that uses either a battery or a hydrogen fuel cell, and there is currently a significant difference between these systems. In the case of a battery system, electrification is possible simply by installing a battery, which provides a method of electrifying the vehicle with a relatively simple part replacement method. However, since the charging process is time-consuming, compared with hydrogen fuel cells, it is difficult to apply battery systems to agricultural tractors. In the case of hydrogen fuel cells, the hydrogen tank charging time is relatively shorter compared with a battery, and it has the advantage of a higher energy density than battery systems [20,21,22]. For this reason, research has been conducted on powertrain systems using hydrogen fuel cell power packs [23,24,25,26,27]. Varlese [23] researched a powertrain using a hydrogen fuel cell power pack. In this study, a test bench for a hydrogen fuel cell power pack was constructed, and research on power pack energy efficiency according to load conditions was conducted. An analysis of cooling and energy efficiency according to test results and research on applying this technology to vehicles were conducted. Moreover, Wang [24] researched power distribution control. A power system using the hydrogen fuel cell power pack, battery, and capacitor was developed, and a load prediction using a prediction model for each component was carried out. A characteristic analysis of each component and a study on a control strategy according to each load condition was also conducted.

As previously mentioned, many studies using hydrogen fuel cell power packs have been conducted. However, all these studies focused on control strategies using simulation and prediction models. The operating time for a tractor using a hydrogen fuel cell power pack should be considered because tractors are vehicles for continuous operation and the operation is conducted under high-load conditions. Accordingly, it is important for the operating time of tractors to be increased by considering the load and efficiency of the powertrain. Therefore, an analysis was conducted using measured load data and powertrain specifications. To evaluate vehicle performance, an efficiency analysis of the designed vehicle’s powertrain was conducted using data measured during agricultural operation. Furthermore, an efficiency performance evaluation was conducted through comparison and an analysis with internal combustion engine tractors of the same level.

## 2. Materials and Methods

### 2.1. Data Analysis Process

**Figure 1:**
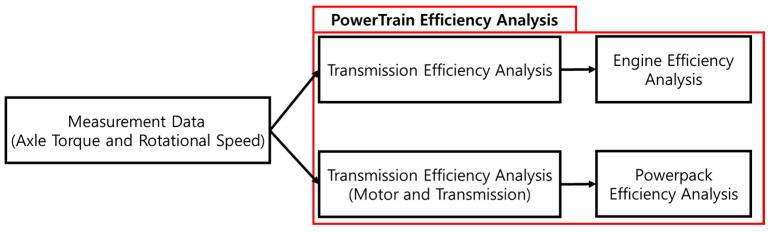
The flow chart of the efficiency analysis for this study.

Measured data using internal combustion engine tractor were used to analyze the powertrain’s efficiency. A powertrain consists of the power source (engine and powerpack) and transmission. In this study, the analysis of the powertrain proceeds as shown in the figure below (Figure 1). The data for the analysis of the powertrain efficiency were measured using the 100 kW class engine tractor. The hydrogen fuel cell power pack tractor is under development; thus, actual vehicle data measurements were impossible. The load was measured using an engine tractor, and this was used for efficiency analyses for both tractors (the engine and the hydrogen fuel cell tractor). The transmission’s efficiency was measured according to transmission specifications, and an analysis of the power source’s efficiency was conducted using measured data (for engine efficiency) and previous research (for hydrogen fuel cell power packs).

### 2.2. Powertrain for Agricultural Tractor

**Figure 2:**
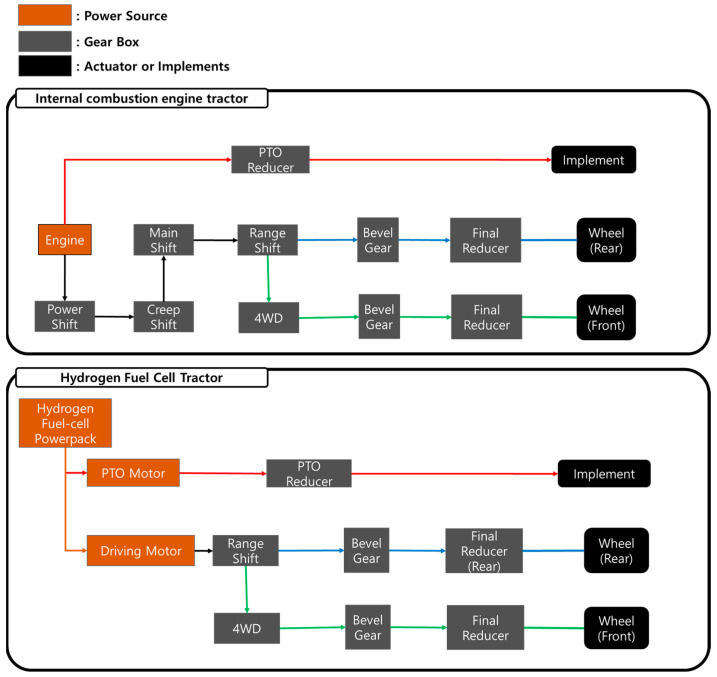
The configurations of the powertrain for the internal combustion engine tractor and hydrogen fuel cell tractor.

The specifications of the transmission for a hydrogen fuel cell tractor and an engine tractor were used in this study. The gear stage of the transmission for an engine tractor was set at different gear ratios to set the speed required for each task. The transmission was analyzed by separating it into plow and rotary operations, as shown in the figure below (Figure 2); therefore, the gear ratio for each operation was set differently as shown in the table below (Table 1).

The powertrain of an internal combustion engine tractor consists of an engine and a transmission. The power of the internal combustion engine tractor is transmitted as follows: first, power is transmitted to the powershift that determines forward and backward motion; then, power is transmitted to the creep stage, which is operated when the driver needs to drive at low speeds. Power is transmitted to the main shift and range shift; then, it is transmitted to a 4WD (wheel drive), bevel gear, and planetary gear set (final reducer) to drive the vehicle. The PTO (power take-off) gearbox has a gear ratio of 4.1, and power is directly connected to the engine. The reduction gear ratio of rear wheel is 156.6, and it is 112.4 for the front wheel with respect to the plow tillage operation. The reduction gear ratio of the rear wheel is 176.7, the front wheel is 126.8, and PTO is 4.1 for the rotary tillage operation. 

The powertrain of a hydrogen fuel cell tractor consists of motors, a transmission system, and hydrogen fuel cell power packs. The two motors, the driving motor and PTO motor, were used for the hydrogen fuel cell power pack tractor. The power from the driving motor is transmitted to the range shift; then, it is transmitted to a 4WD, bevel gear, and planetary gear set (final reducer) to drive the vehicle. The power of the PTO motor is directly connected to the implement. The reduction gear ratio of the rear wheel is 100.8; for the front wheel, it is 70.4; and for the PTO, it is 5.2. The hydrogen fuel cell tractor uses the same gear ratios to carry out both plow and rotary operations.

The analysis of power source efficiencies was conducted using measurement data and considering previous studies. Efficiency was calculated using fuel consumption and engine power for the engine tractor, and the efficiency analysis of the power pack was conducted with reference to previous studies.

#### 2.2.1. Transmission Efficiency Analysis

The power values of the engine, PTO, and axles were calculated using the measured data and the following equation.

(1) $$P=\frac{2\pi TN}{60,000}$$

P is the power of the engine, the PTO, and the axle (kW). T is the torque of the engine, the PTO, and the axle (Nm); N is the rotational speed of the engine, the PTO, and the axle (rpm). The analysis of the transmission for the powertrain was conducted using measured data during agricultural operations. The efficiency of the gear for transmission was equal, and it was considered the same for all products (transmission for hydrogen fuel cell and engine tractors) [25].

(2) $$\beta_{transmission}=\frac{P_{axle}}{P_{axle}+P_{L}}100$$

(3) $${P_{L}=P}_{M_{i}}+P_{W}$$

$\beta_{transmission}$ is the efficiency of the transmission (%), and $P_{Axle}$ is the measured axle power during the agricultural operation for this study. $P_{L}$ is the total transmission power loss (kW), $P_{M_{i}}$ is the mesh power loss of the gear (kW), and $P_{W}$ is the gear windage and churning power loss (kW). The power transmission efficiency is determined by the losses due to the load caused by sliding and rolling motions generated on the tooth surface and the speed loss caused by flow resistance. The power loss of the gear was calculated using ISO standard [28] equations, as shown below.

(4) $$P_{M_{i}}=\frac{f_{m}T_{1}n_{1}{cos}^{2}\beta_{\omega }}{9549M}$$

$f_{m}$ is the mesh coefficient of friction (Nm), $T_{1}$ is the pinion torque (Nm), $\beta_{\omega }$ is the operating helix angle (rad), and M is the mesh mechanical advantage. Here, the power loss due to speed was calculated using the equation below. The power loss due to speed is calculated via the sum of three types of losses: the loss related to the outer diameter of the gear, the loss related to the disk’s surface, and the loss related to the gear tooth surface.

(5) $$P_{W}=P_{GW1}+P_{GW2}+P_{GW3}$$

(6) $$P_{GW1}=\frac{7.37^{*}f_{g}{vn}^{3}D^{4.7}L}{A_{g}10^{26}}$$

(7) $$P_{GW2}=\frac{1.474^{*}f_{g}{vn}^{3}D^{5.7}}{A_{g}10^{26}}$$

(8) $$P_{GW3}=\frac{7.37^{*}f_{g}{vn}^{3}D^{4.7}LF(\frac{R_{f}}{\sqrt{\tan\beta }})}{A_{g}10^{26}}$$

$P_{GW1}$ denotes losses associated with a smooth outside diameter (kW); $P_{GW2}$ denotes losses associated with the smooth sides of a disc (kW); $P_{GW3}$ denotes losses associated with tooth surfaces (kW); $P_{W}$ denotes gear windage and churning power loss (kW); $f_{g}$ denotes the gear dip factor; v denotes the kinematic viscosity of the oil at operating temperatures (cSt); n denotes the rotational shaft speed (rpm); D denotes the outside diameter of the element (mm); $A_{g}$ denotes the arrangement constant; L denotes the length of the element for gearing windage and churning (mm); F denotes the total face width of the gear or pinion (mm); $R_{f}$ denotes the roughness factor for the gear teeth; and β is the generated helix angle (rad). Transmission efficiency calculations were performed based on the above equations and design data.

#### 2.2.2. Power Source Efficiency Analysis

The total capacity of the power required for the engine tractor was calculated using the low diesel calorific value and engine fuel efficiency [26,27]. The equation is as follows:

(9) $$Q_{engine}=E_{engine}\frac{Q_{axle}}{{LCV}_{diesel}}$$

$Q_{Engine}$ denotes the capacity of the power required during agricultural operations considering the efficiency of the engine (kWh). $E_{Engine}$ denotes the fuel consumption efficiency according to the capacity of the power required (L/kWh); it is calculated using Equation (9). $Q_{Axle}$ denotes the capacity of the power required during agricultural operations on the axle; it is calculated using Equation (10). ${LCV}_{Diesel}$ denotes the low calorific value of diesel; it is 10.5 kWh/L [29,30].

(10) $$E_{engine}=\frac{{FC}_{engine}}{P_{engine}}$$

(11) $$Q_{Axle}=\int \frac{T_{axle}}{\beta_{transmission}\gamma_{transmission}}$$

${FC}_{Engine}$ denotes the fuel rate (L/h), and $P_{Engine}$ is the average engine power calculated using measured load data. Engine power was calculated using measured engine torque and rotation speeds during agricultural operations. $T_{Axle}$ is the measured torque data (Nm), and $\gamma_{transmission}$ is the gear ratio on each axle. The total capacity of the power required for the hydrogen fuel cell tractor ($Q_{Powerpack})$ was calculated using the efficiency of the powerpack, motor, and inverter and the capacity of the power required during the axle’s agricultural operations.

(12) $$Q_{Powerpack}=E_{powerpack}E_{motor}E_{inverter}Q_{Axle}$$

$E_{powerpack}$ is the efficiency of the powerpack (%), $E_{motor}$ is the efficiency of the motor, and $E_{inverter}$ is the efficiency of the inverter. The efficiency of the powerpack is 55% [8], the efficiency of the motor is 95%, and the efficiency of the inverter is 95%. 

### 2.3. Agricultural Tractor and Implements

**Figure 3:**
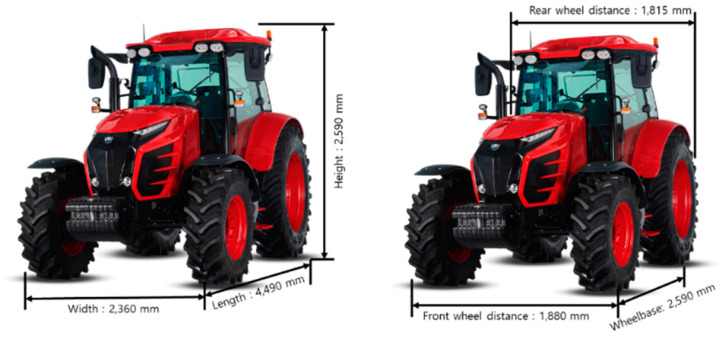
Dimensions of the tractor used in this study.

The tractor (T130, TYM, Iksan, Republic of Korea) used for this study was a 95 kW class tractor (@2200 rpm), and Table 2 lists its detailed specifications. Figure 3 shows the dimensions (length × width × height) of the tractor and the wheel distance (front and rear) and wheelbase. The dimensions of the tractor are 4490 × 2360 × 2940 mm. The wheelbase is 2590 mm, the front and rear wheel distances are 1880 mm and 1815 mm, respectively, and the weight is 4545 kg. The front and rear tires are 380/85R24 and 460/85R38, respectively. The engine is a common rail engine controlled via CAN (controller area network) data.

**Figure 4:**
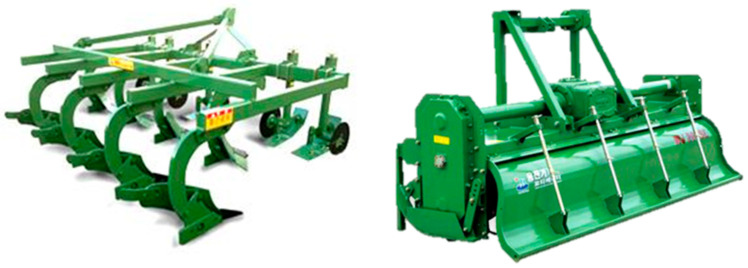
The plow (**left**) and rotary (**right**) tillers used in this study.

Plow tillage and rotary tillage operations were conducted for data measurement; these operations are the most frequently performed operations when using agricultural tractors. Plow and rotary tillage operations were carried out at a tillage depth of about 15–20 cm. Plow and rotary tillage operations were selected as shown in the figure below (Figure 4) considering the power of the tractor, and the width of the implements was selected differently according to the power of the tractor. In Korea, implements with a width of 2.8 m or more for plow tillage and 2.6 m or more for rotary tillage are used for tractors with an engine output of 95 kW. An 8-row plow tiller (WJSP-8, Woong-jin Machinery, Kimje, Republic of Korea) with a width of 2.8 m and a rotary tiller (WJ260C, Woong-jin Machinery, Republic of Korea) with a width of 2.6 m and 9 flanges were used for the measurement test. The length, width and height of the plow tillage are 2180, 2800, and 1285 mm, respectively, and the length, width, and height of the rotary tillage are 900, 2730, and 1250 mm, respectively. 

### 2.4. Measurement System for Agricultural Tractor

**Figure 5:**
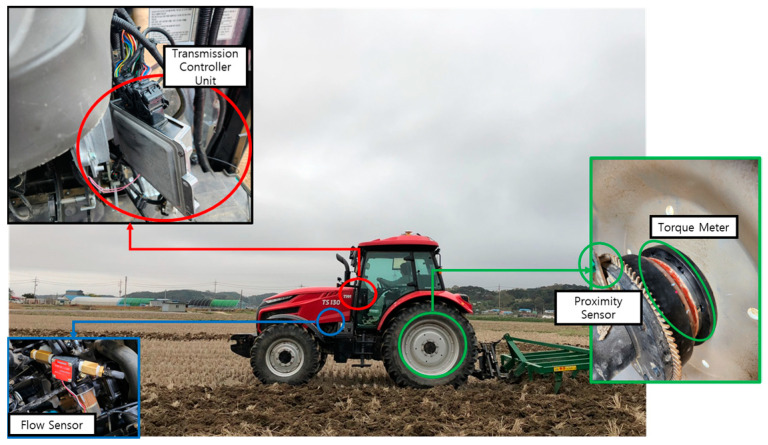
The configuration of the measurement system of the engine tractor for the measurement test.

The measurement system consists of four components as shown in the figure below (Figure 5). First, engine data load measurements are calculated using the vehicle’s CAN data. Second, the axle load is measured using an axle torque meter and proximity sensors. Third, fuel consumption is measured using the sensor installed on the fuel line.

#### 2.4.1. Engine Torque and Speed Measurement

The engine measurement system was configured by connecting the TCU (transmission control unit) and the measuring instrument in parallel. The engine CAN communication of the vehicle used in the test was set to the SAE standard [31]. Since the TCU is connected in parallel with the ECU (engine control unit), the data of the ECU (engine torque and rotational speed) can be measured through parallel connections with the TCU. The TCU controller, which is located in the tractor cabin, and the engine’s CAN data required for this study are as follows: engine torque and rotation speed. The measurement instrument was set using the CAN information provided by J1939, and the data were obtained during agricultural operations.

#### 2.4.2. Axle and PTO Torque and Rotational Speed Measurement

**Figure 6:**
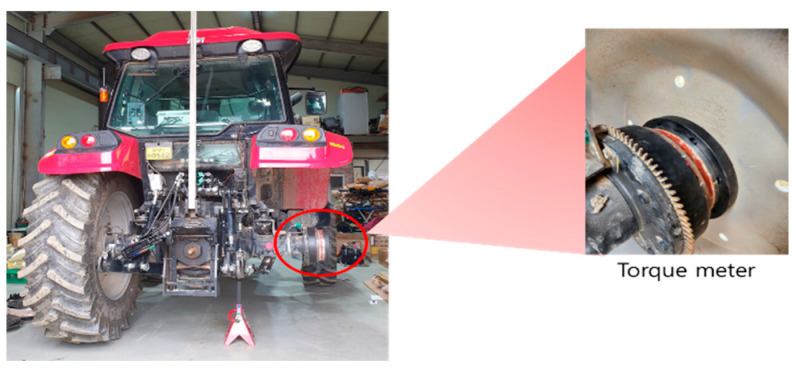
Axle torque meter installed on the tractor’s axle.

The torque meter and proximity sensor were installed on the axle as shown in the figure below (Figure 6), and they were used to measure the torque and the rotational speed generated by the axle. The front-wheel torque meter (MW-15, Manner, Spaichingen, Germany) is capable of measuring up to 15 kNm, and the rear-wheel torque meter (MW-30, Manner, Germany) is capable of measuring up to 30 kNm.

**Figure 7:**
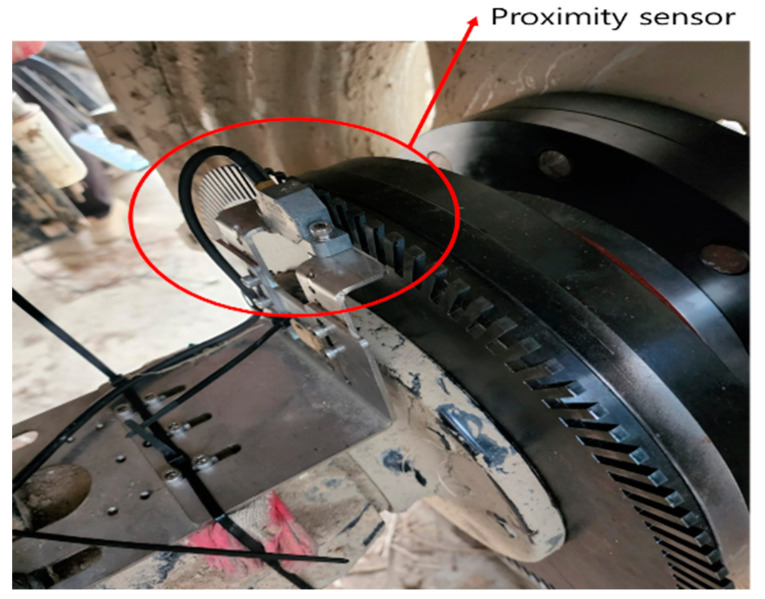
Proximity sensor installed on the axle for axle rotational speed measurements.

Proximity sensors (Pickup Type 8a, Manner, Germany) were installed on the axle as shown in the figure below (Figure 7) to measure the rotational speed by detecting the rotation of the disk.

The PTO load was measured using a PTO torque meter (MK-5kNm, Manner, Germany), which is capable of measuring up to 5 kNm. The torque and rotational speed of the PTO were measured using the torque meter.

#### 2.4.3. Flow Rate Measurement

**Figure 8:**
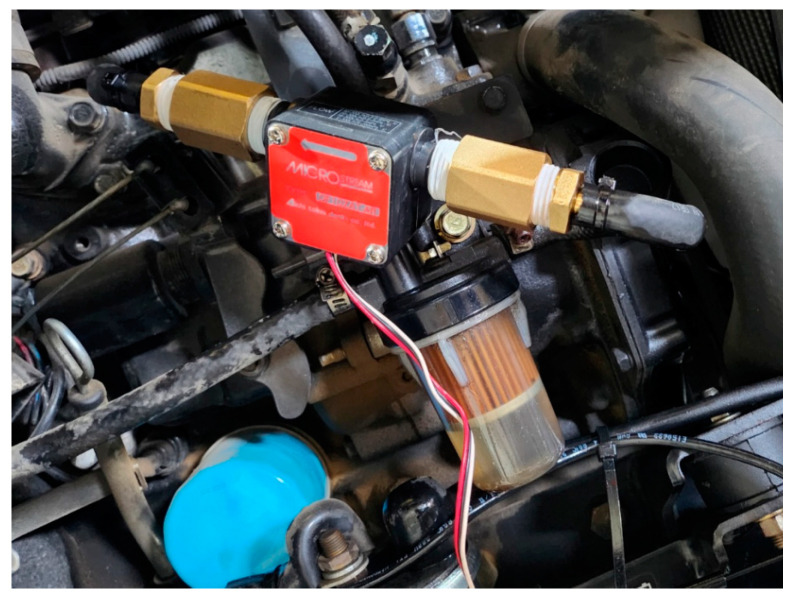
Flow sensor installed on the fuel flow line to measure fuel consumption during agricultural operations.

Fuel consumption was measured by installing a flow sensor (OG-2, Wintech process, Chungju, Republic of Korea). The sensor can measure up to 20 L per minute, and it is installed in parallel on the input and output fuel lines as shown in the figure below (Figure 8) to measure fuel consumption during farming operations.

### 2.5. Measurement Test

**Figure 9:**
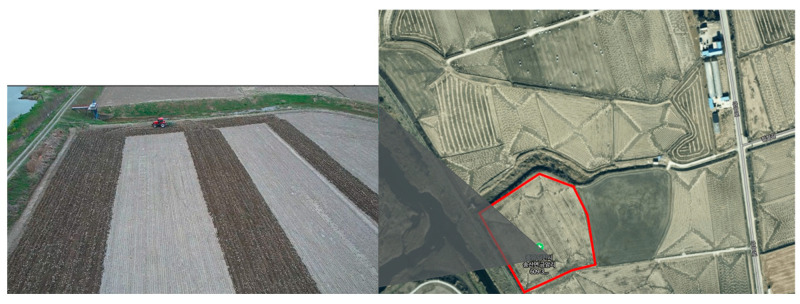
The field where the tractor instrumentation test was conducted.

The load cycle consists of measurement data from an agricultural tractor. The load measurement test for the load cycle was conducted in a paddy field (36.931551, 126.632158) located in Dang-jin, Chungcheongnam-do as shown in the figure below (Figure 9). The field is shown in the picture below; the area of the field is about 6600 m^2^ (100 m × 60 m).

**Figure 10:**
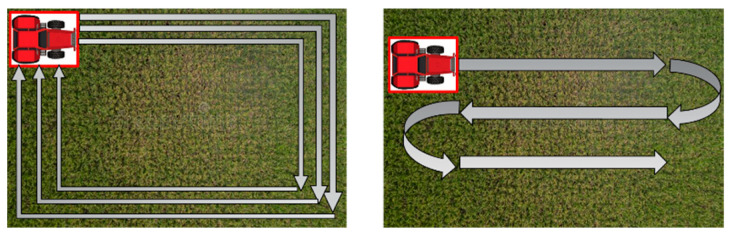
Turning (**left**) and straight (**right**) routes.

The agricultural operation was performed using the same work pattern as farmers who use the field. The operations were largely divided into straight and turning driving routes, as shown in the figure below (Figure 10).

**Figure 11:**
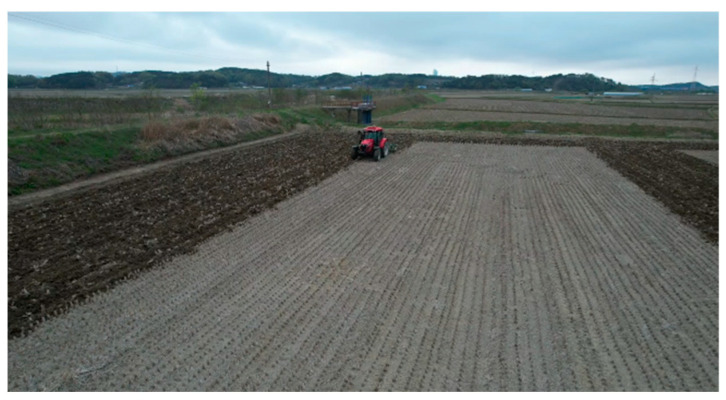
Turing operation pattern used in this study.

In a plow tillage operation, the straight-driving operation was carried out after the turning operation. The turning operation was carried out to prepare the space for the tractor to turn before the straight-driving operation was conducted, as shown in the figure below (Figure 11).

**Figure 12:**
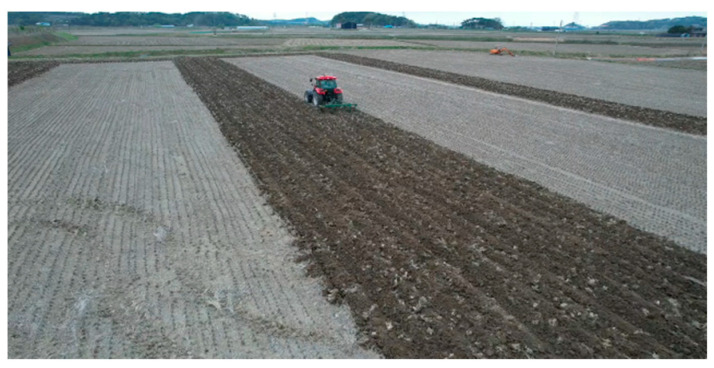
The straight-driving operation pattern used in this study.

The straight-driving operation comprises a pattern that takes up the remaining space after the turning operation is complete, as shown in the figure below (Figure 12). 

**Figure 13:**
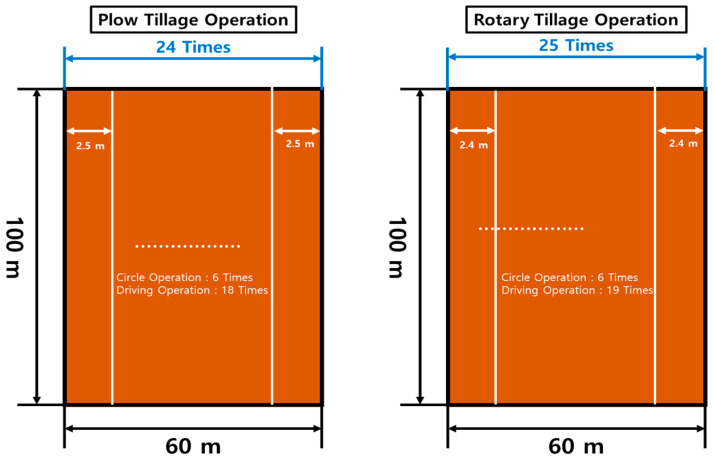
Number of repetitions and test overview according to agricultural operations.

The number of repetitions for the plow and rotary operations was determined as shown in the figure below (Figure 13). The width of the plow tillage was 2.8 m wide, and considering the overlapping section, a total of 24 repetitions could be conducted and measured. The width of the rotary tillage was 2.6 m wide, and considering the overlapping section, a total of 25 repetitions were performed and measured.

The work speed during the agricultural operation was set to 5–7 km/h for the plow tillage operation and 3–5 km/h for the rotary tillage operation. The plow tillage operation was carried out in one field (6600 m^2^) and conducted for about 2770 s. The rotary tillage operation was carried out in the same field as the plow tillage operation was performed, and it was conducted for about 3728 s.

## 3. Results

### 3.1. Measurement Data during the Plow and Rotary Tillage Operations

#### 3.1.1. Engine Power and Fuel Rate

**Figure 14:**
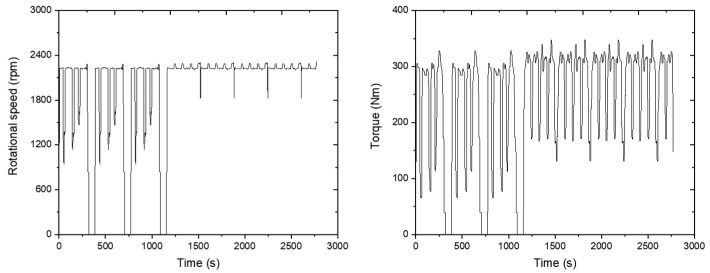
Engine torque (**left**) and rotational speed (**right**) measured during the plow tillage operation.

The plow tillage operation was conducted for 2770 s. The circle operation was repeated six times, and the driving operation was repeated eighteen times. The engine’s rotation speed was around the set 1975 rpm, and the engine torque was 243.7 Nm on average as shown in the figure below (Figure 14).

**Figure 15:**
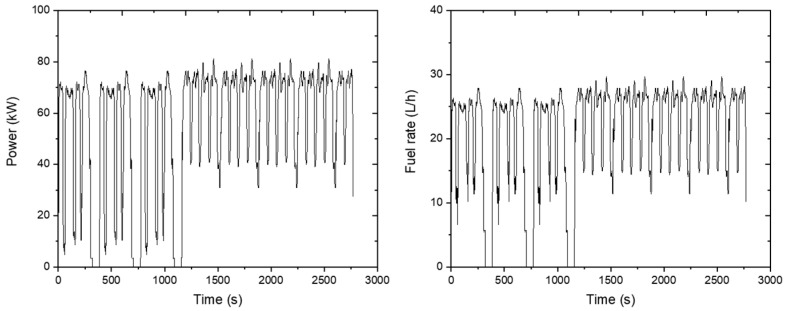
Measured engine power data (**left**) and fuel rate (**right**) during the plow tillage operation.

The load appeared to be constant without large variations during the agricultural operation, and it dropped significantly only in no-load conditions when the tractor turned. This shows that the average engine load was 56.1 kW. Fuel consumption was measured at about 21.1 L/h during plow tillage operations. The results are plotted on a graph as shown below (Figure 15).

#### 3.1.2. Axle Load and Rotational Speed

**Figure 16:**
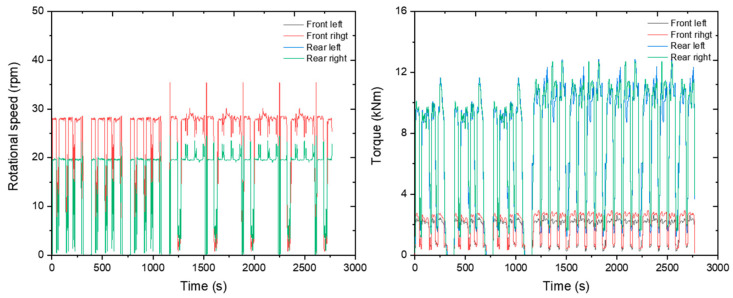
Axle rotational speed (**left**) and axle load (**right**) measured during the plow tillage operation.

The rotational speed of the axle was measured at about 28 rpm for the front wheel and about 20 rpm for the rear wheel. The front axle’s torque was measured at about 1800 Nm, and the rear axle’s torque was about 7400 Nm. The results are plotted on a graph as shown below (Figure 16).

### 3.2. Measurement Data during the Rotary Tillage Operation

#### 3.2.1. Engine Power and Fuel Rate

**Figure 17:**
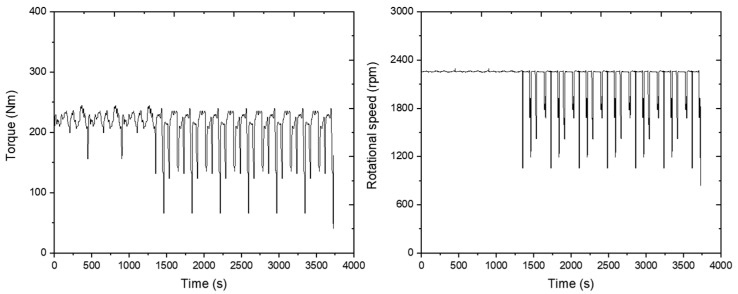
Engine torque (**left**) and rotational speed (**right**) measured during the rotary tillage operation.

The rotary operation was performed for about 3728 s. The circle operation was repeated six times, and the driving operation was repeated nineteen times. The engine’s rotational speed was around the set 2168 rpm, and the engine torque was 208.8 Nm on average. The results are plotted on a graph as shown below (Figure 17).

**Figure 18:**
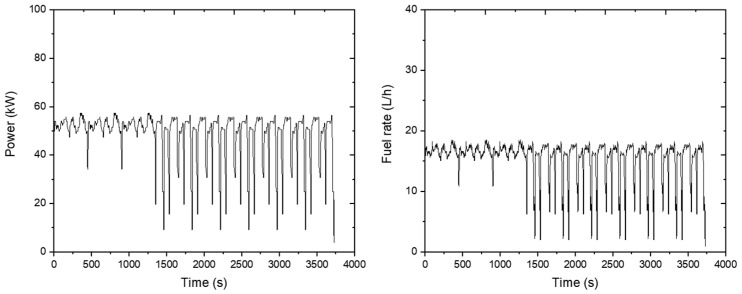
Measured engine power data (**left**) and fuel rate (**right**) during the rotary tillage operation.

The load appeared to be constant and without large variations during the agricultural operation, and it decreased significantly only in no-load conditions when the tractor turned. This shows that the average engine load was 48.6 kW. Fuel consumption was measured at about 15.5 L/h during rotary tillage operations. The results are plotted on a graph as shown below (Figure 18).

#### 3.2.2. Axle Load and Rotational Speed

**Figure 19:**
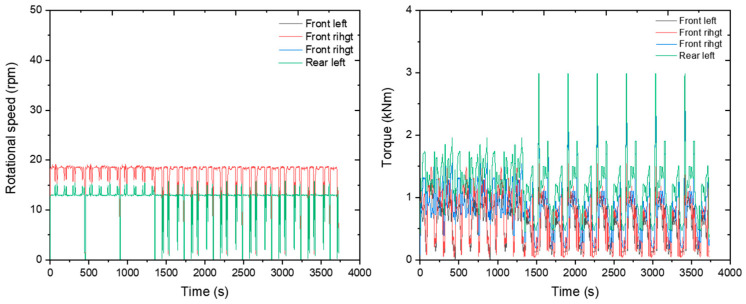
Axle rotational speed (**left**) and axle load (**right**) measured during the rotary tillage operation.

The rotational speed of the axle was measured at about 18 rpm for the front wheel and about 13 rpm for the rear wheel. The front axle’s torque was measured at about 600 Nm, and the rear axle’s torque was about 1200 Nm. The results are plotted on a graph as shown below (Figure 19).

#### 3.2.3. PTO Load Data

**Figure 20:**
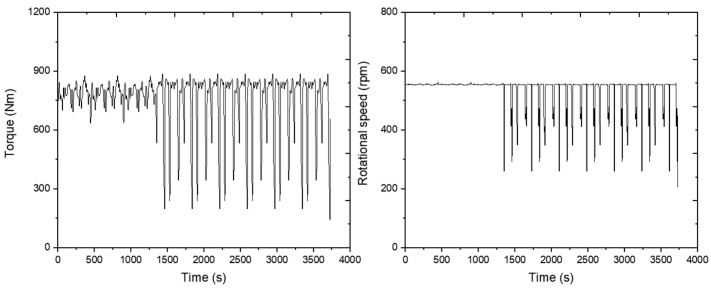
Measured PTO torque (**left**) and rotational speed (**right**) during the rotary tillage operation.

The PTO torque and rotational speed were measured using the PTO torquemeter. The average torque of the measurement data for the PTO was 752 Nm, and the rotational speed was about 532 rpm. The results are plotted on a graph as shown below (Figure 20).

#### 3.2.4. Summary of Measurement Test during Agricultural Operations

The measurement results during agricultural operations are shown in the table below (Table 3). The plow and rotary tillage operations were conducted for 2770 and 3728 s, respectively. The average torque values during the plow and rotary tillage operations were 243.7 and 208.8 Nm, respectively. The engine’s rotational speeds during the plow and rotary tillage operations were 1975 and 2168 rpm, respectively. The average engine power was measured at 56.1 and 48.6 kW, respectively, during plow and rotary tillage operations. Fuel consumption was measured at 29.7 and 15.5 L/h. The front axle’s torque and rotational speeds during plow tillage operations were 1743.6 Nm and 28 rpm, and for the rear axle, they were 7740.8 Nm and 20 rpm, respectively. The front axle’s torque and rotational speed during rotary tillage operations were 628.1 Nm and 18 rpm, and for the rear axle, they were 1216.1 Nm and 13 rpm, respectively.

### 3.3. Transmission Efficiency Analysis Results

The results of the efficiency calculations with respect to the specifications of each gear are shown in the table below (Table 4). In the case of the transmission for the hydrogen fuel cell tractor, it exhibits a high efficiency because the transmission was configured monotonously and was operated via the motor. In the case of the transmission system for the engine tractor, the engine’s rotational speed is fixed; thus, many gear pairs are connected to carry out the agricultural operation. This is because the required speed for each operation is different; thus, different speeds have to be implemented according to each operation. Accordingly, it was determined that multiple gears are connected, and this exhibited low transmission efficiency. The plow tillage operation exhibits higher efficiency than the rotary tillage operation, and this is because the gear windage and churning power loss are lower than the mesh power loss; thus, it is determined that the efficiency is higher in the plowing operation with a higher rotation speed.

The transmission efficiency of the rear and front axles for the plow tillage operation is 87.3 and 85.6%, respectively. The transmission efficiency of the rear and front axle and PTO for the rotary tillage operation is 76.2, 74.7%, and 98.1%, respectively. The transmission efficiency of the rear and front axle and PTO for the hydrogen fuel cell tractor is 92.5, 90.8, and 98.1%.

### 3.4. Powertrain Efficiency Analysis

#### 3.4.1. Engine Fuel Efficiency Analysis

The results of engine fuel efficiency analysis are as shown in the table below (Table 5). The engine’s fuel efficiency analysis was conducted using the averaged fuel rate data and engine power. The engine fuel efficiency analysis for plow and rotary tillage operations is 0.37 L/kWh and 0.32 L/kWh, respectively. A lower efficiency point within the engine was used for plow tillage operations, and this shows that more fuel is used during plow tillage operations. The measured capacity of the required power was calculated using the transmission gear ratio, efficiency, and measured load (axle torque and rotational speed), and the results show that plow and rotary tillage operations were calculated to be 52.1 and 33.8 kWh, respectively. The calculated capacity considering the engine fuel efficiency for plow and rotary tillage operations exhibits 131.2 and 175.1 kWh, respectively.

#### 3.4.2. Hydrogen Fuel Cell Tractor Efficiency Analysis

The results of hydrogen fuel cell tractor efficiency analysis are as shown in the table below (Table 6). The measured capacity of the required power was calculated using the transmission gear ratio, efficiency, and measured load (axle torque and rotational speed), and the results show that the plow and rotary tillage operations were calculated to be 31.2 and 52.1 kWh, respectively. The capacity of the required power, considering the powertrain efficiency for hydrogen fuel cell tractors for plow and rotary tillage operations, was calculated using the efficiency of the motor, inverter, and powerpack, and values of 62.9 and 51.3 kWh were observed.

#### 3.4.3. Comparative Analysis of Efficiencies According to the Power Source

The engine tractor shows lower efficiencies than hydrogen fuel cell power pack tractors because the engine tractors have complex transmission structures and exhibit low fuel efficiency. Considering these factors, the engine exhibited an efficiency of about 47.9% compared to the power pack in the case of the plow tillage operation, and the engine exhibited an efficiency of about 29.3% in the case of the rotary tillage operation. The results of efficiency analysis are shown in the table below (Table 7).

## 4. Conclusions

In this study, power transmission performance analyses using the specifications of recently developed eco-friendly vehicles were carried out. The load (engine torque, engine rotational speed, axle torque, and axle rotational speed) was measured using an engine tractor, and the analysis was conducted using the measured data and specifications of the powertrain. In the case of the engine tractor, the analysis was performed on the engine and transmission. In the case of the hydrogen fuel cell tractor, the analysis was performed using the specifications of the motor, inverter, power pack, and transmission. More fuel was consumed according to the plow tillage operation in the case of the test using the engine because the load variation was lower than the rotary tillage operation. Accordingly, the plow tillage operation exhibited a higher required output even though it was performed for more than 1000 s less than the rotary tillage operation.

The transmission efficiency was calculated using the ISO-14179-1 equation. The transmission of the engine tractor exhibited a lower transmission efficiency than the hydrogen fuel cell power pack powertrain, and this is because it was designed considering the engine’s characteristics and therefore requires many gear trains. To analyze the efficiency of the entire powertrain, an analysis of the power source’s efficiency was conducted. In the case of the hydrogen fuel cell tractor, the efficiency of the power pack was calculated using the specifications of the developed vehicle and not test data. Since the structure is simple in the case of powertrains using hydrogen fuel cell power packs, a high efficiency is exhibited compared to the engine tractor; the efficiency is about 2.1 times greater in the case of the plow tillage operation and about 3.4 times greater in the case of the rotary tillage operation. The hydrogen fuel cell tractor is judged to be suitable for high-efficiency and eco-friendly vehicles because it can use eco-friendly power sources (hydrogen) while utilizing the advantages of motors (high efficiency). In addition, since the charging time is short compared to the electric charging system, the hydrogen fuel cell tractor will be the most suitable for replacing the current diesel engine.

These studies have the following limitations: There are no actual test data for the hydrogen fuel cell power pack, motor, and inverter. Therefore, future studies should conduct individual tests with respect to the hydrogen fuel cell power pack, motor, and inverter, and research should verify the efficiency of the entire vehicle through measurement tests (agricultural operations) using the developed vehicle.

## Figures and Tables

**Table 1:** Gear ratio of transmission according to agricultural operations.

Vehicle	Operation	Gear Set	Reduction Gear Ratio
Internal combustion engine tractor	Plow tillage	Rear axle	156.6
		Front axle	112.4
	Rotary	Rear axle	176.7
		Front axle	126.8
		PTO	4.1
Hydrogen fuel cell tractor		Rear axle	100.8
		Front axle	70.4
		PTO	5.2

**Table 2:** Specifications of the tractor used in this study.

Name	Specification
Engine rated power (kW)	95 (@2200 rpm)
Length (mm)	4490
Width (mm)	2360
Height (mm)	2590
Wheelbase (mm)	2590
Weight (kg)	4545

**Table 3:** Summary of measurement results during agricultural operations.

Average Data		Plow Tillage	Rotary Tillage
Working time (s)		2770	3728
Engine torque (Nm)		243.7	208.8
Engine rotational speed (rpm)		1975	2168
Engine power (kW)		56.1	48.6
Fuel consumption (L/h)		29.7	15.5
Axle torque (Nm)	Front	1743.6	628.1
	Rear	7470.8	1216.1
Axle rotational speed (rpm)	Front	28	18
	Rear	20	13
PTO torque (Nm)		-	752
PTO rotational speed (rpm)			532

**Table 4:** Transmission efficiency analysis results considering gear specifications.

Vehicle	Operation	Gear Set	Transmission Efficiency (%)
Engine tractor	Plow tillage	Rear axle	87.3
		Front axle	85.6
	Rotary	Rear axle	76.2
		Front axle	74.7
		PTO	98.1
Hydrogen fuel cell tractor		Rear axle	92.5
		Front axle	90.8
		PTO	98.1

**Table 5:** Summary of the engine fuel efficiency analysis.

	Plow Tillage	Rotary Tillage
Average data of engine power (kW)	56.1	48.6
Average data of fuel consumption (L/h)	21.1	15.5
Engine efficiency (L/kWh)	0.37	0.32
The measured capacity of required power (kWh)	33.8	52.1
Fuel consumption (L)	16.7	12.5
The calculated capacity considering the engine fuel efficiency (kWh)	131.2	175.1

**Table 6:** Summary of the hydrogen fuel cell tractor efficiency analysis.

	Plow Tillage	Rotary Tillage
The measured capacity of required power (kWh)	31.2	31.2
The calculated capacity considering the powertrain efficiency for hydrogen fuel cell tractors (kWh)	62.9	51.3

**Table 7:** Summary of efficiency analysis by type of agricultural tractor.

Operation	Vehicle	The Calculated Capacity (kWh)	Efficiency of the Engine Tractor (%)
Plow	Engine	131.2	47.9
	Hydrogen fuel cell	62.9	
Rotary	Engine	175.1	29.3
	Hydrogen fuel cell	51.3	

## Data Availability

The original contributions presented in the study are included in the article.
